# A comparison of probable post-traumatic stress disorder and alcohol consumption among active female members of the UK Police Service and UK Armed Forces

**DOI:** 10.1007/s00127-022-02356-1

**Published:** 2022-09-07

**Authors:** Patricia Irizar, Sharon A. M. Stevelink, David Pernet, Suzanne H. Gage, Neil Greenberg, Simon Wessely, Laura Goodwin, Nicola T. Fear

**Affiliations:** 1 Department of Sociology, School of Social Sciences, Faculty of Humanities, University of Manchester, Oxford Road, Manchester, M13 9PL UK; 2 Institute of Psychiatry, Psychology and Neuroscience, King’s Centre for Military Health Research, King’s College London, London, UK; 3 King’s Centre for Military Health Research, Institute of Psychiatry, Psychology & Neuroscience, King’s College London, London, UK; 4 The Wellcome Trust, London, UK; 5 The Spectrum Centre for Mental Health Research, Division of Health Research, School of Health and Medicine, Lancaster University, Lancaster, UK; 6 Academic Department of Military Mental Health, Institute of Psychiatry, Psychology and Neuroscience, King’s College London, London, UK

**Keywords:** Harmful alcohol use, Mental health, Military, Police, Post-traumatic stress disorder

## Abstract

**Background:**

The British Police Service and Armed Forces are male-dominated occupations, characterised by frequent trauma exposure and intensive demands. Female police employees and military personnel may have unique experiences and face additional strains to their male counterparts. This analysis compared the levels of post-traumatic stress disorder (PTSD), hazardous/harmful alcohol consumption, and comorbidity in female police employees and military personnel.

**Methods:**

Police data were obtained from the Airwave Health Monitoring Study (*N* = 14,145; 2007–2015) and military data from the Health and Wellbeing Cohort Study (*N* = 928; phase 2: 2007–2009 and phase 3: 2014–2016). Multinomial/logistic regressions analysed sample differences in probable PTSD, hazardous (14–35 units per week) and harmful (35 + units per week) alcohol consumption, and comorbid problems. We compared covariate adjustment and entropy balancing (reweighting method controlling for the same covariates) approaches.

**Results:**

There were no significant differences in probable PTSD (police: 3.74% vs military: 4.47%) or hazardous drinking (police: 19.20% vs military: 16.32%). Female military personnel showed significantly higher levels of harmful drinking (4.71%) than police employees (2.42%; Adjusted Odds Ratios [AOR] = 2.26, 95% Confidence Intervals [CIs] = 1.60–3.21), and comorbidity (1.87%) than police employees (1.00%, AOR = 2.07, 95% CI = 1.21–3.54). Entropy balancing and covariate-adjustments obtained the same results.

**Conclusions:**

Comparable levels of probable PTSD were observed, which are slightly lower than estimates observed in the female general population. Future research should explore the reasons for this. However, female military personnel showed higher levels of harmful drinking than police employees, emphasising the need for alcohol interventions in military settings.

## Introduction

The Police and Armed Forces are occupations characterised by frequent trauma exposure, and intensive demands, comprising mostly of men. Women constitute approximately 40% of the United Kingdom (UK) Police Service (30% of police officers and 60% of police staff) [1], whereas only 10% of the UK Armed Forces are women [2]. Female police employees and military personnel may have unique experiences and face additional strains to their male counterparts, such as being a minority group, failed belongingness and lack of peer support [3, 4]. Moreover, mental health problems are more common in women in the general population, than in men [5], though the machismo culture of both occupations may lead to the concealment of mental health problems to prove ones toughness and worthiness to be there. Therefore, it is important to investigate the mental health of women in two high-risk occupational groups, and to make comparisons across the occupations, as one group may show greater levels of poor mental health and require additional support.

Historically, alcohol has been used by both occupational groups to enhance unit cohesion or to relieve stress [6, 7]. This could relate to the machismo culture encouraging heavy drinking to increase bonding, regardless of gender [8, 9]. Men and women in the UK Armed Forces show higher rates of hazardous and harmful drinking, compared to men and women in the UK general population [10]. Though women report lower levels of consumption compared to men, in both the Armed Forces and the general population [3, 10]. For UK police employees (sworn officers and non-sworn staff), data from the Airwave Health Monitoring Study showed that 40% of men and 19% of women met criteria for hazardous or harmful drinking [11], with separate data for the UK general population finding that 24% of men and 11% of women met the same criteria (not a direct comparison) [12]. Given that the machismo culture contributes to heavy drinking in these occupations, it is critical to investigate the levels of drinking specifically in women, and to make comparisons between the two occupations, as our analysis of male military personnel and police employees showed much higher rates of harmful drinking in military personnel [13].

In the UK, military women have previously been restricted in their roles, with all combat roles only becoming open to women within recent years (since the data for the current analysis was collected) [3]. Nevertheless, military women are still exposed to trauma, for example, through participating in combat-support operations and serving as combat medics [14]. Police women are not limited in the roles they can obtain, though a larger proportion of women (approx. 60%) are non-sworn police staff (e.g., administration, response call operators, intelligence analysts) than sworn police officers (approx. 30%) [1]. Of the limited literature available, police women are more likely to report physical strains than men, but show no difference in the level of poor mental health [15].

Post-traumatic stress disorder (PTSD) is characterised by intrusive thoughts, increased arousal, and avoidance of reminders, following a traumatic experience [16]. Both occupations are frequently exposed to trauma, increasing the risk of PTSD. Hazardous or harmful alcohol use often co-occur with PTSD [17], as alcohol is used, through avoidance coping, to relieve negative affective states resulting from symptoms [18]. Recent statistics from the UK adult general population (including those economically inactive) show that those with probable PTSD have three times greater odds of reporting hazardous or harmful alcohol use, compared to those without PTSD [19]. However, there is a lack of research into PTSD and hazardous/harmful drinking, and their comorbidity, in female members of the UK Police Service and Armed Forces, and there are no direct comparisons of women in these occupations.

This study aims to compare the levels of (i) probable PTSD, (ii) hazardous and harmful alcohol use, and (iii) comorbid PTSD and hazardous/harmful alcohol use, in a sample of female military personnel and a sample of female police employees. To increase comparability across the samples, this study will explore the impact of a statistical reweighting method, which balances the samples on pre-specified covariates (i.e., year of data collection, age, educational attainment), compared to regression adjustments of the same covariates.

## Materials and methods

### Study samples and data collection

#### Airwave health monitoring study

Data for the police sample were obtained from the Airwave Health Monitoring Study, which is a cohort study of UK police officers and staff, originally designed to determine the long-term health impact of Terrestrial Trunked Radio (TETRA) usage, but obtaining data on a range of additional demographic, occupational, physical/mental health, and lifestyle variables. The study procedure has been described in detail in previous publications [11, 20].

**Fig. 1:**
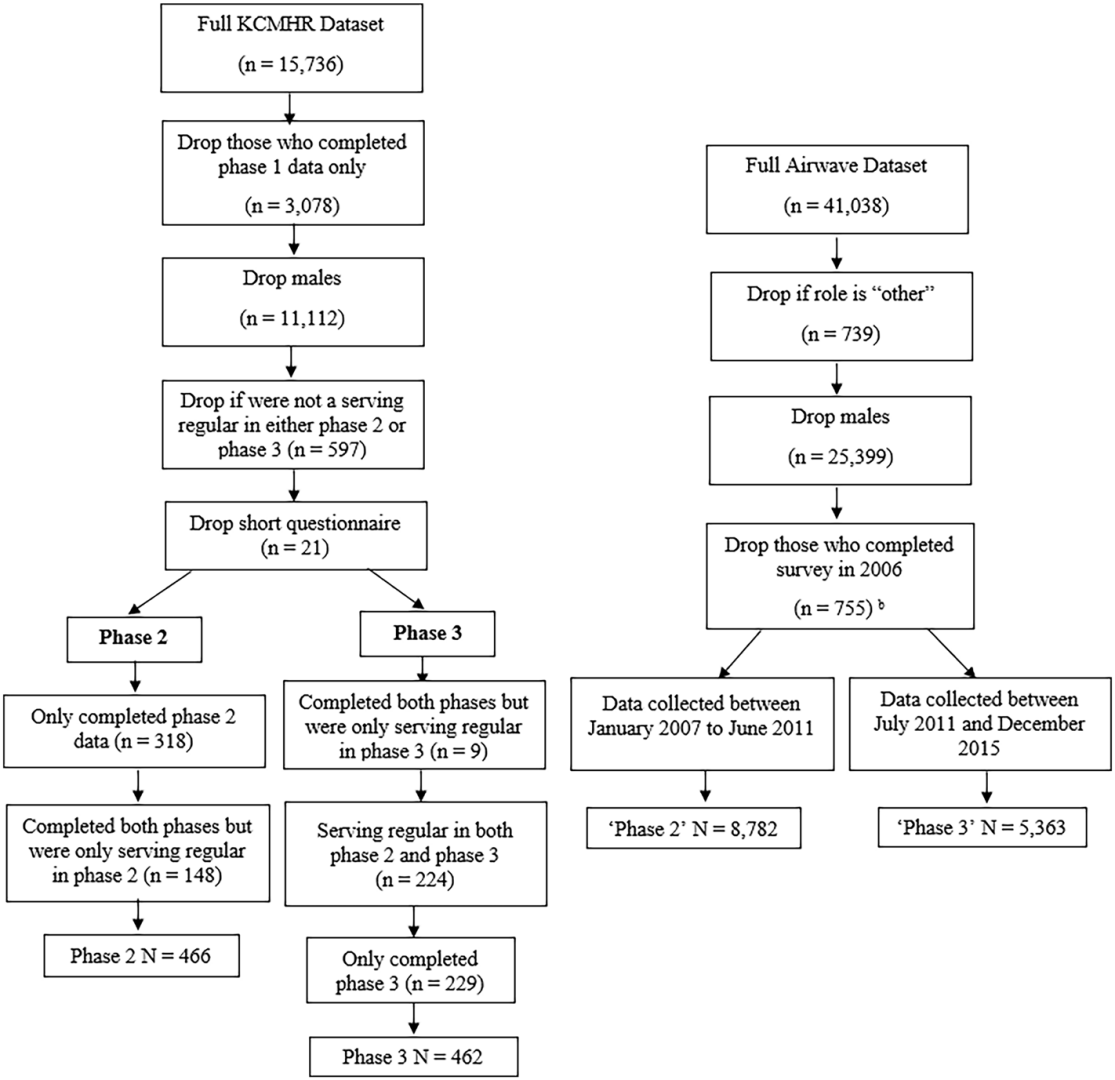
Flow diagram showing the allocation of participants to one category of the binary variable, ‘year of data collection’. Military personnel were allocated to either phase 2 or phase 3. Police employees were allocated to a category which reflected the data collection period from phase 2 or phase 3

Cross-sectional data were collected via an enrolment questionnaire and a health screen, conducted by trained nurses (June 2006–March 2015) for 53,163 police employees from 28 participating police forces across the UK (England, Scotland, Wales). The response rate averaged 50% across participating forces [20]. Out of these participants, 41,038 police employees completed the main outcome variables required for this analysis. Males were excluded from the current analysis as these findings have been reported separately [13], as were those who completed the survey in 2006, as they were not administered the Trauma Screening Questionnaire (TSQ) (Fig. 1). The final sample included 14,145 female police employees.

#### Health and wellbeing cohort study

Data for the military sample were selected from the Health and Wellbeing of UK Armed Forces cohort study, which was established in 2003 to determine the physical and mental health impact of deployment to Iraq (phase 1) [21], then Afghanistan (phase 2) [22], and since to explore the long-term impact of deployment (phase 3) [23]. Data from all three phases were collected via a self-administered questionnaire which was available in hard copy (and an electronic version for phase 3) [21–23]. The questionnaire collected data on the following factors: sociodemographic, service information, experiences during/transitioning from deployment, physical/mental health and lifestyle.

Phase 1 collected data from a random stratified sample of 10,272 personnel (59% response rate; June 2004–March 2006) [21], but participants with only phase 1 data were excluded from this analysis, to increase comparability with the police sample, which did not collect data before 2006. Phase 2 followed-up participants who completed phase 1 (November 2007–September 2009), and obtained data from two additional samples, including a random sample of personnel who had deployed to Afghanistan between April 2006 and April 2007, and a replenishment sample to include personnel who had joined since 2004 (*N* = 9990, 56% response rate). Phase 3 (October 2014–December 2016) collected data from participants who consented to further contact in phases 1 and 2 and from a replenishment sample of new recruits (joined after June 2009), with a final sample size of 8093 personnel (58% response rate) [23].

All phases recruited regular and reservist personnel and those who were still serving and who had left service. To increase comparability with the police sample (which included only serving police employees), only serving regular personnel were included in this analysis. Males were excluded, as were those who completed only phase 1 and who completed the short questionnaire, leaving a total of 928 female military personnel (Fig. 1).

### Measures

#### Demographic, occupational and health variables

Both samples had comparable data on the following demographic and health variables: age (< 30, 30–39, ≥ 40), marital status (married/cohabiting, divorced/separated, single), educational attainment (GSCE/O levels or below, Vocational/A levels or higher), and smoking status. For marital status, police employees had an additional option of “other”, which was re-coded as missing, due to a lack of information on what this included, meaning it could not be compared with the military sample. Data on police role (police staff, police constable/sergeant, inspector or above) were available from the police sample, and military rank (other, non-commissioned officer, commissioned officer) from the military sample.

#### Post-traumatic stress disorder (PTSD)

For police employees, probable PTSD was measured using the ten-item Trauma Screen Questionnaire (TSQ) [24], whereby responses were provided on a five-point scale ranging from ‘not at all’ (scored as 0) to ‘extremely’ (all other responses coded as 1), with total scores of 6 or more being indicative of probable PTSD (range 0–10). However, participants were only asked the PTSD items if they responded ‘yes’ to being bothered by a disturbing incident which occurred in the past 6 months (13% responded ‘yes’).

For military personnel, probable PTSD was measured using the 17-item PTSD Checklist-civilian version (PCL-C) [25], with response options provided on a 5-point scale ranging from ‘not at all’ to ‘extremely’. Scores of 50 or more indicate probable PTSD (range 17–85). Despite different measures being used, previous research has shown that the PCL-C and TSQ obtain similar prevalence estimates for PTSD in the general population [5].

#### Alcohol consumption

In both samples, participants were asked if they currently drink alcohol, with those responding ‘no’ being categorised as ‘non-drinkers’. For police employees, hazardous and harmful alcohol consumption was measured using a past week’s drinks diary, i.e., the total number of drinks from the following options: white wine, red wine, fortified wine, spirits, beer/cider. This was converted to units and categorised into hazardous use (> 14–35 units) and harmful use (> 35 units), based on the UK Chief Medical Officer’s guidelines for low-risk drinking (0–14 units) and the NICE guidelines for hazardous (referred to as ‘increasing risk’) and harmful drinking (referred to as ‘high risk’) specifically for females [26, 27].

The full ten-item Alcohol Use Disorder Identification Test (AUDIT) [28] was administered in the military sample. To increase comparability, only two items were used (frequency of consumption and typical units on a drinking day) to estimate average weekly units, by multiplying frequency (conservative estimate) with the midpoint for typical units (e.g., frequency of two or three times a week = 2, typical units of 3 or 4 drinks = 3.5, would equate to 7 weekly units) [13]. Responses to ‘typical units’ are usually on a five-point scale, up to 10 or more drinks, but additional options were available (up to 30 or more). Hazardous and harmful alcohol consumption was categorised using the same cut-offs as the police sample.

### Statistical analysis

#### Estimating sample differences

All analyses were conducted in STATA SE 15 [29]. Descriptive statistics, i.e., frequencies and proportions, with 95% confidence intervals (CIs), were obtained for the demographic, occupational, and health variables, and for the outcome variables (probable PTSD, categories of alcohol consumption, comorbidity).

Logistic regressions were used to estimate sample differences in probable PTSD (non-case PTSD as reference group). Multinomial logistic regressions were used to estimate differences in the categories of alcohol consumption (non-drinkers, hazardous, harmful, with low risk as reference group, due to evidence indicating a “U” or “J-shaped” relationship between consumption and mental/physical health, whereby both non-drinking and hazardous/harmful drinking are associated with adverse health outcomes, compared to low risk [30, 31]), and to estimate differences in comorbid PTSD and hazardous/harmful alcohol use (presence of neither as reference group). Analyses were adjusted for pre-determined covariates [13] which may affect the outcomes, in steps, first adjusting for year of data collection, age, and educational attainment, then also for marital status and smoking status. The police sample was the reference group as it was the larger sample. Unadjusted and adjusted odds ratios (OR) and multinomial odds ratios (MOR), with 95% CIs are reported.

#### Entropy balancing

Sensitivity analyses explored the impact of entropy balancing and determined whether the estimates from the entropy balanced analyses differed to the regression analyses adjusting for covariates. Entropy balancing is a multivariate reweighting method which creates a weight value for participants in the larger sample to be more comparable to the smaller sample on a range of pre-determined covariates (year of data collection, age, and educational attainment—the second step of adjustments from the previous analyses) [32]. A binary variable was created for year of data collection, to reflect phase 2 (January 2007–June 2011 in the police sample) and phase 3 (July 2011–December 2016 in the police sample) of the military sample. Age was categorised into ‘ < 30 years old’, ‘30–39 years old’, and ‘ ≥ 40 years old’, as there were few female military personnel above 40 years old. Educational attainment was categorised as low (GCSEs/equivalent or below) and high (A levels/equivalent or higher). Entropy balancing was conducted in STATA SE 15 using the *ebalance* command [33]. The regressions estimating sample differences were repeated, now applying the entropy balancing weights, and adjusting for marital status and smoking status.

### Ethics

All participants provided written informed consent. The Airwave Health Monitoring Study received ethical approval from the National Health Service multi-site research ethics committee (MREC/13/NW/0588). The Health and Wellbeing Cohort Study Ethical obtained ethical approval for each phase of from both the UK Ministry of Defence Research Ethics Committee and the local Ethics Committee at King’s College London.

## Results

### Sample characteristics

The sample characteristics are shown in Table 1. The study sample included 14,145 female police employees and 928 female military personnel (total *N* = 15,073). There were large differences in the age distribution between the samples, with 47.20% of the police sample being over 40 years old compared with 14.12% of the military sample. Approximately 70% of both samples were married or cohabiting. Around two thirds of both samples reported high educational attainment. There were more current smokers in the military sample (16.85%), than the police sample (11.24%).

**Table 1:** Demographic (age, marital status, education, income), occupational (role, rank) and health (smoking status) characteristics from police (*N* = 14,145) and military personnel (*N* = 928)

Characteristic	Police				Military			
	Total	*N*	%	95% CI	Total	*N*	%	95% CI
Age (years)	14,145				928			
< 30		2479	17.53	16.91–18.16		447	48.17	44.96–51.39
30–39		4989	35.27	34.49–36.06		350	37.72	34.65–40.89
≥ 40		6677	47.20	46.38–48.03		131	14.12	12.02–16.51
Marital status	13,582				918			
Married/cohabiting		9519	70.09	69.31–70.85		631	68.74	65.66–71.66
Divorced/separated		2625	19.33	18.67–20.20		229	24.95	22.25–27.85
Single		1438	10.59	10.08–11.12		58	6.32	4.91–8.09
Education	14,063				910			
Low (GSCE/O level or below)		4257	30.27	29.52–31.04		330	36.26	33.20–39.45
High (vocational/A levels or higher)		9806	69.73	68.96–70.48		580	63.74	60.55–68.80
Smoking status	14,134				920			
Non-smoker		12,546	88.76	88.23–89.27		765	83.15	80.59–85.44
Current smoker		1588	11.24	10.73–11.77		155	16.85	14.56–19.41
Role (police only)	12,663							
Police staff		6156	48.61	47.74–49.49		–	–	–
Police constable/sergeant		6079	48.01	47.14–48.88		–	–	–
Inspector or above		428	3.38	3.08–3.71		–	–	–
Rank (military only)					928			
Other		–	–	–		205	22.09	19.53–24.88
Non-commissioned officer		–	–	–		471	50.75	47.53–54.97
Commissioned officer		–	–	–		252	27.16	24.39–30.11

### Sample differences in probable PTSD and alcohol consumption

A total of 3.74% of police employees met criteria for probable PTSD, compared with 4.47% of military personnel, and the difference was not statistically significant. Female military personnel were significantly more likely to meet criteria for harmful alcohol consumption (4.71%) than female police employees (2.42%), remaining statistically significant after all adjustments (Adjusted Odds Ratio [AOR] = 2.26, 95% Confidence Intervals [CIs] = 1.60 to 3.21). Military personnel were significantly less likely to be non-drinkers (7.45%) than police employees (11.88%), which remained significant after adjustments (AOR = 0.53, 95% CI = 0.41 to 0.70). Before adjustments, hazardous drinking was significantly lower in military personnel (16.32%) than police employees (19.20%), but this attenuated after adjustments.

Due to small numbers, hazardous and harmful alcohol consumption were combined when estimating sample differences in comorbidity. Of those reporting hazardous/harmful alcohol use, 4.63% (*N *= 140) of female police employees and 8.85% (*N* = 17) of female military personnel also met criteria for probable PTSD. Of those meeting criteria for probable PTSD, 26.62% (*N* = 140) of police employees and 42.50% (*N* = 17) of military personnel also reported hazardous/harmful alcohol use. A four-category variable was created to examine sample differences in comorbid PTSD and hazardous/harmful alcohol (versus meeting criteria for neither). Before and after adjustments, military personnel were significantly more likely to meet criteria for comorbidity (1.87%) than police employees (1.00%; AOR = 2.07, 95% CI = 1.21 to 3.54) (Table 2).

**Table 2:** Logistic and multinomial logistic regressions showing the differences in PTSD, categories of alcohol consumption, and co-occurrence, among police and military personnel

Outcome variable	Police *N* (%)	Military *N* (%)	OR (95% CI)	AOR (95% CI)^a^	AOR (95% CI)^b^
PTSD					
Non-case	13,532 (96.26)	876 (95.53)	1.00	1.00	1.00
Case	526 (3.74)	41 (4.47)	1.20 (0.87–1.67)	1.30 (0.93–1.82)	1.29 (0.92–1.81)
			*N* = 14,975	*N* = 14,946	*N* = 14,448
Alcohol use (UK government guidelines for women)					
Non-drinker	1666 (11.88)	68 (7.45)	0.58 (0.45–0.75)***	0.53 (0.41–0.70)***	0.53 (0.41–0.70)***
Low risk (0–14 units)	9329 (66.50)	653 (71.52)	1.00	1.00	1.00
Hazardous (15–35 units)	2694 (19.20)	149 (16.32)	0.79 (0.66–0.95)*	0.90 (0.74–1.09)	0.90 (0.74–1.09)
Harmful (above 35 units)	339 (2.42)	43 (4.71)	1.81 (1.31–2.51)***	2.30 (1.63–3.25)***	2.26 (1.60–3.21)***
			*N* = 14,941	*N* = 14,877	*N* = 14,382
Co-occurrence					
PTSD non-case and non-case hazardous/harmful alcohol use	10,571 (75.60)	693 (76.32)	1.00	1.00	1.00
PTSD case only	386 (2.76)	23 (2.53)	0.91 (0.59–1.39)	1.00 (0.65–1.56)	1.01 (0.65–1.57)
Hazardous/harmful alcohol use only	2886 (20.64)	175 (19.27)	0.92 (0.78–1.10)	1.08 (0.91–1.29)	1.08 (0.90–1.29)
PTSD case and hazardous/harmful alcohol use	140 (1.00)	17 (1.87)	1.85 (1.11–3.08)*	2.12 (1.24–3.62)**	2.07 (1.21–3.54)**
			*N* = 14,891	*N* = 14,863	*N* = 14,368

The police sample is the reference group

***p < 0.001, **p < 0.01, *p < 0.05

^a^Adjusted for age, education, and year of data collection

^b^Adjusted for marital status and smoking status, in addition to age, education, and year of data collection

### Sensitivity analyses using entropy balancing weights

Table 3 shows the sample characteristics of female police employees before and after the entropy balancing weights were applied. The entropy balancing gave more weight to police employees aged under 30 and less weight to those aged over 40, reflecting the age composition of females in the military sample. The reweighting also resulted in more weight given to police employees who were divorced or separated, a small increase in current smokers, and a small decrease in higher ranking police employees (inspector or above).

**Table 3:** Demographic (age, marital status, education), occupational (role) and health (smoking status) for female police personnel before and after entropy balancing (reweighted based year of data collection, age and educational attainment) (*N* = 14,145)

Characteristic	Police (representative estimates)			Police (entropy balanced)	
	*N*	%	95% CI	%	95% CI
Age (years)					
< 30	2479	17.53	16.91–18.16	50.79	49.48–52.10
30–39	4989	35.27	34.49–36.06	33.12	32.05–34.21
≥ 40	6677	47.20	46.38–48.03	16.08	15.52–16.67
Marital status					
Married/cohabiting	9519	70.09	69.31–70.85	67.31	66.02–68.58
Divorced/separated	2625	19.33	18.67–20.20	26.41	25.18–27.67
Single	1438	10.59	10.08–11.12	6.28	5.78–6.81
Education					
Low (GSCE/O level or below)	4257	30.27	29.52–31.04	36.26	34.91–37.64
High (vocational/A levels or higher)	9806	69.73	68.96–70.48	63.74	62.36–65.09
Smoking status					
Non-smoker	12,546	88.76	88.23–89.27	86.55	85.55–87.49
Current smoker	1588	11.24	10.73–11.77	13.45	12.51–14.45
Role (police only)					
Police staff	6156	48.61	47.74–49.49	45.37	43.99–46.75
Police constable/sergeants	6079	48.01	47.14–48.88	53.19	51.81–54.57
Inspector or above	428	3.38	3.08–3.71	1.44	1.26–1.64

The results of the sensitivity analyses are presented in Table 4. The entropy balancing marginally reduced the proportion of police employees meeting criteria for probable PTSD and harmful alcohol use, which may indicate reduced likelihood of these outcomes in younger female police employees (as more weight is given to younger participants). After applying the entropy balancing and adjusting for marital status and smoking status, the significant differences and effect sizes (odds ratios) remained similar, showing no difference in the level of probable PTSD but higher levels of harmful alcohol use and comorbidity in military personnel compared to police employees.

**Table 4:** Sensitivity analyses, reweighting the police sample using entropy balancing. Logistic and multinomial regression analyses showing the differences in PTSD, categories of alcohol consumption, and co-occurrence, among police and military personnel

Outcome variable	Police *N* (%)	Military *N* (%)	OR (95% CI)	AOR (95% CI)^a,b^
PTSD				
Non-case	13,532 (96.64)	876 (95.53)	1.00	1.00
Case	526 (3.36)	41 (4.47)	1.37 (0.98–1.93)	1.34 (0.95–1.89)
			*N* = 14,946	*N* = 14,448
Alcohol use (UK government guidelines for women)				
Non-drinker	1666 (12.61)	68 (7.45)	0.54 (0.41–0.71)***	0.54 (0.41–0.71)***
Low risk (0–14 units)	9329 (68.45)	653 (71.52)	1.00	1.00
Hazardous (15–35 units)	2694 (17.07)	149 (16.32)	0.91 (0.75–1.11)	0.92 (0.75–1.11)
Harmful (above 35 units)	339 (1.88)	43 (4.71)	2.44 (1.71–3.48)***	2.37 (1.65–3.42)***
			*N* = 14,877	*N* = 14,382
Co-occurrence				
PTSD non-case and non-case hazardous/harmful alcohol use	10,571 (78.56)	693 (76.32)	1.00	1.00
PTSD case only	386 (2.49)	23 (2.53)	1.07 (0.68–1.67)	1.05 (0.67–1.64)
Hazardous/harmful alcohol use only	2886 (18.07)	175 (19.27)	1.10 (0.92–1.32)	1.09 (0.91–1.31)
PTSD case and hazardous/harmful alcohol use case	140 (0.88)	17 (1.87)	2.22 (1.29–3.83)**	2.14 (1.24–3.71)**
			*N* = 14,863	*N* = 14,368

The police sample is the reference group

***p < 0.001, **p < 0.01, *p < 0.05. Percentages are weighted with entropy balancing (year of data collection, age and educational attainment)

^a^Age, education were not adjusted for as these variables were used in the entropy balancing

^b^Adjusted for marital status and smoking status

## Discussion

### Key findings

This was the first study to directly compare the level of probable PTSD, hazardous and harmful alcohol consumption, and their comorbidity, between female military personnel and police employees. The level of probable PTSD was comparable across the samples, as was the level of hazardous drinking. However, female military personnel were more likely to meet criteria for harmful alcohol use and less likely to be non-drinkers, than female police employees. Military women also showed higher levels of comorbid PTSD and hazardous/harmful alcohol use, though the levels were small in both samples and most likely driven by the higher levels of harmful alcohol use in military women. To increase confidence in the findings, as there were large demographic differences between the samples, this study compared two statistical techniques to control for the same pre-specified covariates (regression adjustment and entropy balancing), finding that covariate adjustment had the same impact as entropy balancing on the level of association and effect sizes.

### Sample differences

The comparable levels of probable PTSD in female military personnel and police employees are harmonious with the findings from our analysis of male military personnel and police employees (approximately 4% for men and women in both occupational groups) [13]. This is consistent with previous evidence, whereby female gender is a risk factor for PTSD in general population samples, but studies of police and military samples often show no difference between genders [34]. The levels of PTSD are slightly lower than observed in women in the general population (5.1%), especially women aged 16–24 years old, who show much higher levels of PTSD (12.6%) [5]. Almost half of the military sample were under 30 years old, whereas almost half of the police sample were over 40 years old, suggesting lower levels of probable PTSD in young military women compared to young women in the general population. However, a direct comparison with data from the general population, controlling for age, is needed.

Given the high levels of trauma exposure in these occupational groups, higher levels of PTSD may have been expected. In addition, experiences of sexual trauma and assault (prevalence reported to be approximately 30% among military women [14]) may further elevate the risk. Nevertheless, this is in line with previous literature, showing reduced PTSD symptom severity in police employees, compared to civilians, following a traumatic incident [35]. The relatively low levels of PTSD across both men and women in these occupations could reflect protective effects of leadership, training, and support [36]. Another explanation may be the healthy worker effect [37], as longitudinal evidence shows higher levels of mental health problems in military personnel who have left service, compared to those still in service [23]. Those with poorer mental health may leave service [38], or alternatively, ex-serving personnel may have reduced access to support and services or experience difficulties transitioning to civilian life [39]. When focussing on women in these occupations, the low levels of PTSD could be related to protective characteristics of women who enter male-dominated environments, such as self-assertion, toughness, and resilience [40], or it may be that the machismo culture within these occupations leads to the concealment of poor mental health. Future research should also monitor police employees’ mental health after they leave service to determine whether additional support is needed.

As with the analysis of males, the present study also identified higher rates of harmful drinking, and comorbidity, in female military personnel, compared to female police employees [13]. The levels of harmful drinking observed in male and female police employees (approximately 3% for both) are similar to general population estimates (4% of men and 3% of women, using the same criteria) [12], but are higher in both military men (10%) and women (5%), in line with an existing direct comparison [10]. The higher levels of harmful drinking in military women may relate to the male-dominated drinking culture being more prominent in the Armed Forces than the Police Service, as settings which facilitate drinking are still available in the military (e.g. messes) but no longer exist for police employees (e.g. removal of police bars) [6, 7]. Although the proportion of those meeting criteria for both conditions was low in the whole population, a large proportion of those with probable PTSD were drinking at hazardous/harmful levels (43% of military personnel, 27% of police employees), suggesting that women in both occupational groups may be using alcohol to cope. Alternatively women may show high-levels of help-seeking and receive adequate support if needed [41].

### Strengths and limitations

This study utilised large samples of female military personnel and police employees, with moderate response rates (above 50%). Given that both occupations are male-dominated, women are often underrepresented when researching these occupations. This study is the first to directly compare the levels of probable PTSD and hazardous/harmful alcohol use in female military personnel and police employees, and separately from men. By taking a robust approach to increase comparability, first adjusting for covariates in regression models and then applying weights to balance the samples on the same covariates, we have provided robust estimates of sample differences. However, there are several limitations. The main limitation is the difference in measures used. Though we were able to harmonise the measures of alcohol consumption, the PTSD measures were not directly comparable. For example, the 17-item PCL-C was used in the military sample and the 10-item TSQ was used in the police sample with the latter being limited to participants who had been bothered by a traumatic incident in the 6 months prior and the former having no restrictions regarding when the trauma occurred. There are currently no formal comparisons of the TSQ and PCL-C, so the validity of this approach is unknown. However, a general population survey used the PCL-C in the most recent wave and the TSQ in previous waves, obtaining similar prevalence estimates for PTSD [5]. Nevertheless, as only 13% of the police sample completed the TSQ, the prevalence of PTSD in this group may be underestimated, preventing the identification of statistically significant differences between the two samples. The three-item AUDIT was used in the military sample and a weekly drinks diary was used in the police sample, however, the additional response options in the military sample (up to 30 drinks or more) allowed the identification of those drinking too much higher levels (unlike the traditional AUDIT which provides a more restricted level of consumption as the highest response option is 10 or more drinks), as with the drinks diary. Furthermore, due to the smaller sample size and small number of female military personnel meeting criteria for probable PTSD and harmful drinking, we were not able to explore the demographic associations with these outcomes, as with the analysis of males [13]. We were also unable to control for important intersecting factors, such as ethnicity, which was not recorded in the military sample, despite cultural differences and experiences of discrimination being important for understanding differences in mental health and alcohol use. Finally, the use of self-report measures may mean that alcohol consumption was underestimated [42, 43], though self-report measures were used for both samples and so any bias or effect is likely to apply to both data collections.

### Implications

Previous research has shown that unit cohesion and good leadership are protective against probable PTSD in military research [36], and future research should explore whether similar factors are protective in police employees and other high-risk occupations. The higher levels of harmful alcohol use observed in military women supports existing evidence and emphasises the need for alcohol-reduction interventions, such as digital apps which are currently being trialled [44], tailored specifically for military personnel of both genders.

### Conclusions

This study identified comparable levels of probable PTSD in female military personnel and police employees, which are slightly lower than estimates in the female general population, but higher levels of harmful alcohol use were observed in military women compared to policewomen; reasons for this should be explored.

## Data Availability

The police employee data underlying the results presented in the study are available to researchers who apply for access to the Airwave Health Monitoring Study via the following URL: https://police-health.org.uk/applying-access-resource. The military personnel data are not publicly available.
